# Immune response and innervation signatures in aseptic hip implant loosening

**DOI:** 10.1186/s12967-016-0950-5

**Published:** 2016-07-07

**Authors:** Daniel M. Vasconcelos, Manuel Ribeiro-da-Silva, António Mateus, Cecília Juliana Alves, Gil Costa Machado, Joana Machado-Santos, Diogo Paramos-de-Carvalho, Inês S. Alencastre, Rui Henrique, Gilberto Costa, Mário A. Barbosa, Meriem Lamghari

**Affiliations:** i3S - Instituto de Investigação e Inovação em Saúde, Universidade do Porto, Porto, Portugal; INEB - Instituto de Engenharia Biomédica, Universidade do Porto, Porto, Portugal; ICBAS - Instituto Ciências Biomédicas Abel Salazar, Universidade do Porto, Porto, Portugal; Serviço de Ortopedia e Traumatologia, Centro Hospitalar São João, Porto, Portugal; Faculdade de Medicina, Universidade do Porto, Porto, Portugal; Instituto Português de Oncologia do Porto, Porto, Portugal

## Abstract

**Background:**

Aseptic loosening (AL) of hip prosthesis presents inflammation and pain as sign and symptom similarly to arthritis pathologies. Still, the immune and innervation profiles in hip AL remain unclear and their interplay is poorly explored. Herein, local tissue inflammatory response, sensory and sympathetic innervation as well as associated local mediators were assessed in hip joint microenvironment underlying AL and compared to osteoarthritis (OA).

**Methods:**

Histopathological analysis, immune cells (macrophages, T, B cells and PMNs) as well as sensory and sympathetic nerve fibers (SP^+^, CGRP^+^, TH^+^) distribution and profiles were analyzed on tissues retrieved from patients with failed hip prostheses due to AL (n = 20) and hip OA (n = 15) by immunohistochemistry. Additionally, transcriptional levels of pro-inflammatory cytokines (TNF-α, IL-1β, IL-6, IL-12a, iNOS), anti-inflammatory cytokine (IL-10), osteoclastic factor (RANKL) and bone remodeling factor (TGF-β1) were locally evaluated by qRT-PCR. Serum TGF-β1 levels were assessed preoperatively by ELISA.

**Results:**

Histopathological analysis revealed that tissues, aseptic interface membranes of AL patients had distinct tissue architecture and immune cells profile when compared to OA synovial tissues. Macrophages, T cells and B cells showed significant differences in tissue distribution. In OA, inflammation is mostly confined to the vicinity of synovial membrane while in AL macrophages infiltrated throughout the tissue. This differential immune profile is also accompanied with a distinct pattern of sensory and sympathetic innervation. Importantly, in AL patients, a lack of sympathetic innervation aseptic interface membranes without compensation mechanisms at cellular levels was observed with simultaneous reorganization of sensorial innervation. Despite the different histopathological portrait, AL and OA patients exhibited similar transcriptional levels of genes encoding key proteins in local immune response. Nevertheless, in both pathologies, TGF-β1 expression was prominent in sites where the inflammation is occurring. However, at systemic level no differences were found.

**Conclusion:**

These findings indicate that AL patients exhibit different local inflammatory response and innervation signatures from OA patients in hip joint. These insights shed the light on neuro-immune interplay in AL and highlight the need to better understand this crosstalk to unravel potential mechanisms for targeted-therapies to improve hip joint lifetime and treatment.

**Electronic supplementary material:**

The online version of this article (doi:10.1186/s12967-016-0950-5) contains supplementary material, which is available to authorized users.

## Background

Osteoarthritis (OA) has long been considered a cartilage driven “wear and tear” disease that may lead to hip joint failure [1, 2]. The pain and diminished hip joint motion induced by OA may be effectively treated through primary hip replacement [3, 4]. Unfortunately, hip replacement is not a permanent solution as prostheses often fail 15–25 years after primary surgery, mostly due to aseptic loosening (AL), infection and dislocation [5, 6]. Other therapeutic solutions are then urgently required to expand implants’ lifetime.

Inflammation is common to AL and OA [1, 7, 8]. Immune cells and cytokines have been previously reported in both clinical scenarios [8–11]. Synovial inflammation is often observed in OA patients and is frequently characterized by the infiltration of specific immune cell populations, such as macrophages, T cells and mast cells, as well as by the expression of pro-inflammatory cytokines, as Tumor necrosis factor-α (TNF-α) and interleukin-1β (IL-1β) [7]. Nevertheless, these findings were mostly described in studies focusing on the knee or in studies combining data from both knee and hip, without anatomical discrimination [7]. On the other hand, periprosthetic joint inflammation is commonly known as a complex local biological response that takes place on synovial membrane-like interface tissues, triggered by implant released by-products (particles and ions) [12–15]. Of note, the nature, size and amount of released particles are recognized to define the inflammatory profile [13–16]. While polymeric particles such as polyethylene (PE) and polymethylmethacrylate (PMMA) are often associated with macrophage mediated foreign body reaction and tissue fibrosis, high levels of metallic particles and ions have been demonstrated to promote tissue necrosis and lymphocyte-driven responses [17].

Pain is one of the clinical features observed in both AL and OA conditions [18, 19]. Innervation profile is mostly studied in the context of arthritic diseases but not in the presence of implantable biomaterials. Patients and animal studies highlighted innervation of synovial tissues as a possible player in the pain process and, based on anatomical mapping, suggested a coupling of innervation and inflammation in osteoarthritic synovial tissues [20, 21]. It is well documented that both sympathetic, tyrosine-hydroxylase (TH)^+^ or neuropeptide Y (NPY)^+^, and sensory nerve fibers, substance P (SP)^+^ and calcitonin gene related peptide (CGRP)^+^, are present in OA synovial tissue and grow towards cartilage along blood vessels [21]. On the other hand, so far, two studies addressed the innervation of the interface membranes surrounding AL hip prostheses [19, 22]. Unfortunately, the data is still unclear. Niissalo et al. reported that the synovial membrane-like interface did not contain C-sensory peptidergic or sympathetic neural structures while Ahmed et al. identified sympathetic nerve fibers in these interface membranes [19, 22]. Thus, innervation profile and its possible association with periprosthetic joint inflammation, triggered by prosthetic debris in AL scenario, should be revised. Furthermore, the comparison of the inflammatory versus innervation profiles of AL and OA has never been examined and requires further investigation. In this study, the immune and innervation profiles of AL and OA were addressed dissecting local players at tissue, cellular and molecular levels with potential systemic translation.

## Methods

### Patients and samples

Centro Hospitalar São João ethics committee approved this study and all patients consented to the use of their tissue and blood for research purposes. The followed procedures were in accordance with the Helsinki Declaration of 1975, as revised in 2000. Samples from synovial membrane-like interface tissue/aseptic interface membrane were collected from twenty patients during hip revision surgeries due to AL of hip prostheses. All revised hips had a metal-on-polyethylene (MoP) coupling, eleven out twenty cemented and bone defects were classified according to the Paprosky classification [23]. Relevant clinical information is summarized in Table 1. Infection, recurrent dislocation and periprosthetic fracture were considered exclusion criteria in the aseptic loosening group. Osteoarthritic synovial tissues were collected from 15 patients undergoing primary hip replacement surgeries for primary OA. OA patients were classified for OA severity according Tönnis OA grade [24] and presented scores from moderate (2) to severe (3). The clinical information of these patients is summarized in Table 2. For both OA and AL groups, the same orthopedic team performed the collection of tissue samples. Previously to surgery, blood was collected and leukograms and plain radiographies were registered.

**Table 1:** Clinical data from aseptic loosening patients

Aseptic loosening case number	Age	Gender	Hip prostheses type	Implant fixation	Time to revision (months)	Component revised	Type of bone defect	Metallosis
1	79	M	MoP	Cemented	13	Acetabular	2B	
2	61	F	MoP	Cemented	24	Acetabular	2A	
3	74	F	MoP	Cemented	23	Acetabular	2B	
4	86	F	MoP	Cemented	46	Acetabular	2B	
5	50	F	MoP	Uncemented	56	Acetabular femoral	3A1	Yes
6	79	F	MoP	Cemented	96	Acetabular	3A	
7	73	M	MoP	Cemented	108	Acetabular	3A	
8	69	F	MoP	Cemented	117	Acetabular	2C	
9	74	M	MoP	Cemented	120	Acetabular femural	2A1	
10	77	M	MoP	Uncemented	120	Acetabular femoral	2B1	
11	45	F	MoP	Uncemented	129	Acetabular	3A	
12	73	F	MoP	Uncemented	130	Acetabular	1	Yes
13	63	F	MoP	Cemented	138	Acetabular	3A	
14	75	M	MoP	Cemented	144	Acetabular	3A	
15	62	F	MoP	Uncemented	153	Acetabular	2C	
16	53	F	MoP	Uncemented	156	Acetabular	2A	
17	86	F	MoP	Cemented	168	Acetabular femoral	2A1	
18	71	F	MoP	Uncemented	204	Acetabular	1	
19	82	F	MoP	Uncemented	216	Acetabular	1	Yes
20	75	M	MoP	Uncemented	240	Acetabular	1	Yes

**Table 2:** Clinical data from osteoarthritis patients

Osteoarthritis case number	Age	Gender	OA severity (tonnis classification)
1	79	F	2
2	45	F	3
3	54	F	3
4	80	F	3
5	55	M	3
6	49	F	2
7	76	M	3
8	51	F	2
9	56	F	3
10	71	M	3
11	74	F	3
12	83	M	3
13	37	F	3
14	74	M	3
15	66	M	2

Immediately after excision, both OA synovial tissues and aseptic interface tissues were split. Half of the tissue was immersed in formalin for further histological analysis, while dry ice was used to freeze the remaining tissue until storage at −80 °C. No more than 4 h elapsed between tissue collection and storage.

### Histochemistry and immunostaining

Half of collected tissues were formaldehyde-fixed paraffin embedded and cross-sections of 3 μm thickness were cut. Contiguous sections were stained with hematoxylin & eosin (H&E) and Masson’s trichrome (MT). For immunohistochemistry, tissue sections were deparaffinized and rehydrated before heat induced antigen retrieval (98 °C, 10 mM citrate buffer, pH 6.0). Endogenous peroxidases were blocked using 3 % H_2_O_2_ and non-specific binding sites were blocked using Background Block (Cell Marque, USA), previously to the incubation with antibody diluent 1 % BSA (negative control) or with primary antibodies: anti-CD20 (clone L26, dilution 1:100, Cell Marque, USA), anti-CD163 (clone MRQ-26, dilution 1:150, Cell Marque, USA), anti-CD68 (clone 514H12, dilution 1:100, Novocastra, UK), anti-HLA-DR (clone TAL1B5, dilution 1:5000, Abcam, USA) and anti-CD3 (clone PS1, dilution 1:100, Biocare Medical, USA). After primary antibody incubation, tissue sections were incubated with BrightVision Poly-HRP-Anti Mouse/Rabbit/Rat IgG (Immunologic, the Netherlands) and then revealed using DAB Plus Substrate System (Thermo Scientific, USA), before hematoxylin counterstaining. The specificity of immunostainings of CD20, CD163, CD68, HLA-DR and CD3 was confirmed using human spleen as positive control. For immunofluorescence studies, tissue section were deparaffinized and antigen retrieval of rehydrated sections was performed using Proteinase K (0.2 mg/mL in PBS) for NF200 immunostaining and incubated for 20 min at 98 °C in Citrate buffer (pH 6.0), or TE Buffer (pH 9.0) for SP and CGRP staining, respectively. After quenching endogenous fluorescence with 0.1 % sodium borohydride and 100 mM NH_4_Cl, sections were incubated with blocking buffer (10 % FBS, 1 % BSA, 0.2 % Triton X-100). Primary antibody rabbit anti-human neurofilament heavy subunit (NF200) (dilution 1:1000, Abcam, USA), anti-Substance P (dilution 1: 1000, Millipore, USA), anti-TH (dilution 1:100, Millipore, USA), anti-CGRP (dilution 1:4000, Sigma-Aldrich, USA) or blocking buffer (negative control) was applied overnight at 4 °C. For signal detection, tissue sections were incubated with anti-rabbit Alexa Fluor 568 antibody (dilution 1:1000, Life Technologies, USA), incubated with DAPI and then mounted with Fluoroshield Mounting Medium (Abcam, USA). The specificity of immunostainings for NF200, Substance P, TH and CGRP was confirmed using a specimen of human Morton’s neuroma as positive control.

### Semi-quantitative histopathological evaluation

In order to characterize synovial microenvironment, tissues were semi-quantified considering the total immunoreactive area and scored into four categories: absent (0), present (1), frequent (2) or abundant (3), according to the histological grading system illustrated in the Additional file 1: Figure S1 (tissue reaction), Additional file 2: Figure S2 (prosthetic debris accumulation) and Additional file 3: Figure S3 (immune cells distribution), similarly to the methodology followed by others [25, 26].

Structural changes, fibrosis and necrosis in OA synovial tissues and aseptic interface tissues were evaluated after H&E and MT staining. The thickening/hyperplasia and increased villi of synovial membrane were assessed in OA patients. The accumulation of polymeric, ceramic and metallic particles was assessed in synovial membrane-like interface tissues. A polarized filter was used to detect polymeric particles. Ceramic and metallic particles were studied through scanning electron microscopy (SEM) and their elemental composition assessed by energy dispersive spectroscopy (EDS). Additionally, a phase contrast filter (Ph3) was used with an optical microscope to ease the detection of clusters of ceramic or metallic particles in histological slices, allowing a deep study of the interaction between particles and cells. The infiltration of target immune cell populations, identified by immunohistochemistry, was determined regarding the presence of macrophages (CD68^+^ cells), T cells (CD3^+^ cells) and B cells (CD20^+^ cells). The semi-quantification of polymorphonucleated cells (PMNs) was performed after PMNs identification as these cells have a lobed nucleus while multinucleated giant cells (GC) were detected due to their bigger size, multiples nucleus and CD68^+^ labeling.

Tissue samples retrieved from five out thirty-five patients (4/15 OA and 1/20 AL) were excluded from histopathological evaluation because they do not correspond to OA synovial tissue or aseptic interface tissue. H&E, MT and immunohistochemistry slices were analyzed using a light microscope Olympus CX31 while immunofluorescence, polarized light and phase contrast Ph3 images were captured on Carls Zeiss Axiovert 200 inverted microscope.

### Gene expression analysis

Synovial tissues were homogenized in liquid nitrogen using a mortar and pestle to preserve RNA integrity. RNA was extracted and purified using TRIzol (Invitrogen, UK) and Direct-zol™ RNA MiniPrep (ZYMO Research, USA), according to the manufacturers’ instructions. The amount and quality of extracted RNA were evaluated by Nanodrop ND-1000 (Thermo Fisher Scientific, USA) and running RNA samples in a 2 % agarose gel. The transcriptional levels of pro-inflammatory cytokines (TNF-α, IL-1β, IL-6, IL-12a and iNOS), anti-inflammatory cytokine (IL-10), osteoclastic factor (RANKL) and bone remodeling factor (TGF-β1) were evaluated by quantitative real time PCR (qRT-PCR) in PCR iQ™5 system (Bio-rad, USA). All used primers, listed in Table 3, were optimized and melting curves of PCR products were evaluated to guarantee primers specificity. β2 microglobulin (B2 M) and β-actin were used as reference genes. Experiments were performed in triplicated. Relative gene expression levels were calculated using the quantification cycle (C_q_) method, according to MIQE guidelines [27]. Fourteen out thirty-six cases (5/15 OA and 9/20 aseptic loosening) were excluded from gene expression analysis when average Cq for reference genes was above 26, to avoid bias in the evaluation of genes with later expression.

**Table 3:** List of primers used for quantitative qRT-PCR analysis

Gene	GenBank number	Forward primer sequence (5′–3′)	Reverse primer sequence (5′–3′)
B2M	[NM_004048]	CCAGCGTACTCCAAAGATTCAG	AGTCAACTTCAATGTCGGATGG
β-actin	[NM_001101]	TACCTCATGAAGATCCTCA	TTCGTGGATGCCACAGGAC
IL-1β	[NM_000576]	CTTCAGCCAATCTTCATT	CACTGTAATAAGCCATCAT
IL-6	[NM_000600]	CAATCTGGATTCAATGAGGAGACT	CTGTTCTGGAGGTACTCTAGGTAT
IL-10	[NM_000572]	GGAGAACCTGAAGACCCTCA	TATAGAGTCGCCACCCTGAT
IL-12a	[NM_000882]	TACCAGGTGGAGTTCAAG	GTTCTTCAAGGGAGGATTT
iNOS	[NM_000625]	AATTGAATGAGGAGCAGGTC	TCCTTCTTCGCCTCGTAA
TNF-α	[NM_000594]	TCTCTCTAATCAGCCCTCTG	TGCTACAACATGGGCTACAG
RANKL	[NM_003701]	GGATGGCTCATGGTTAGA	CAAGAGGACAGACTCACTT
TGF-β1	[NM_000660]	CCTGGACACCAACTATTG	CTTGCGGAAGTCAATGTA

### ELISA

Serum transforming growth factor β1 (TGF-β1) concentrations were measured using Quantikine^®^ ELISA Kit for human TGF-β1 (R&D Systems, USA), according to the manufacturer’s protocol. Cytokine concentration was calculated against a standard curve.

### Statistical analysis

Statistical analysis was performed using SPSS 21.0 (SPSS Inc. Chicago, IL, USA). The level of significance was set at p < 0.05 (*). Visual histogram analysis and Kolmogorov–Smirnov test were used to evaluate the normal distribution of continuous variables (gene expression data, TGF-β1 plasma concentration and percentage of immune cells in blood). Accordingly, these variables were analyzed using Student’s *t* test or its non-parametric counterpart, Mann–Whitney test. Tissue immune response and innervation was histologically classified in different grading categories and comparisons between aseptic interface tissues and OA synovial were done using Chi square test. All graphs were prepared using Prism software (GraphPad software, San Diego, CA, USA).

## Results

### Local immune responses in OA synovial tissues and synovial membrane-like interface tissue

**Fig. 1:**
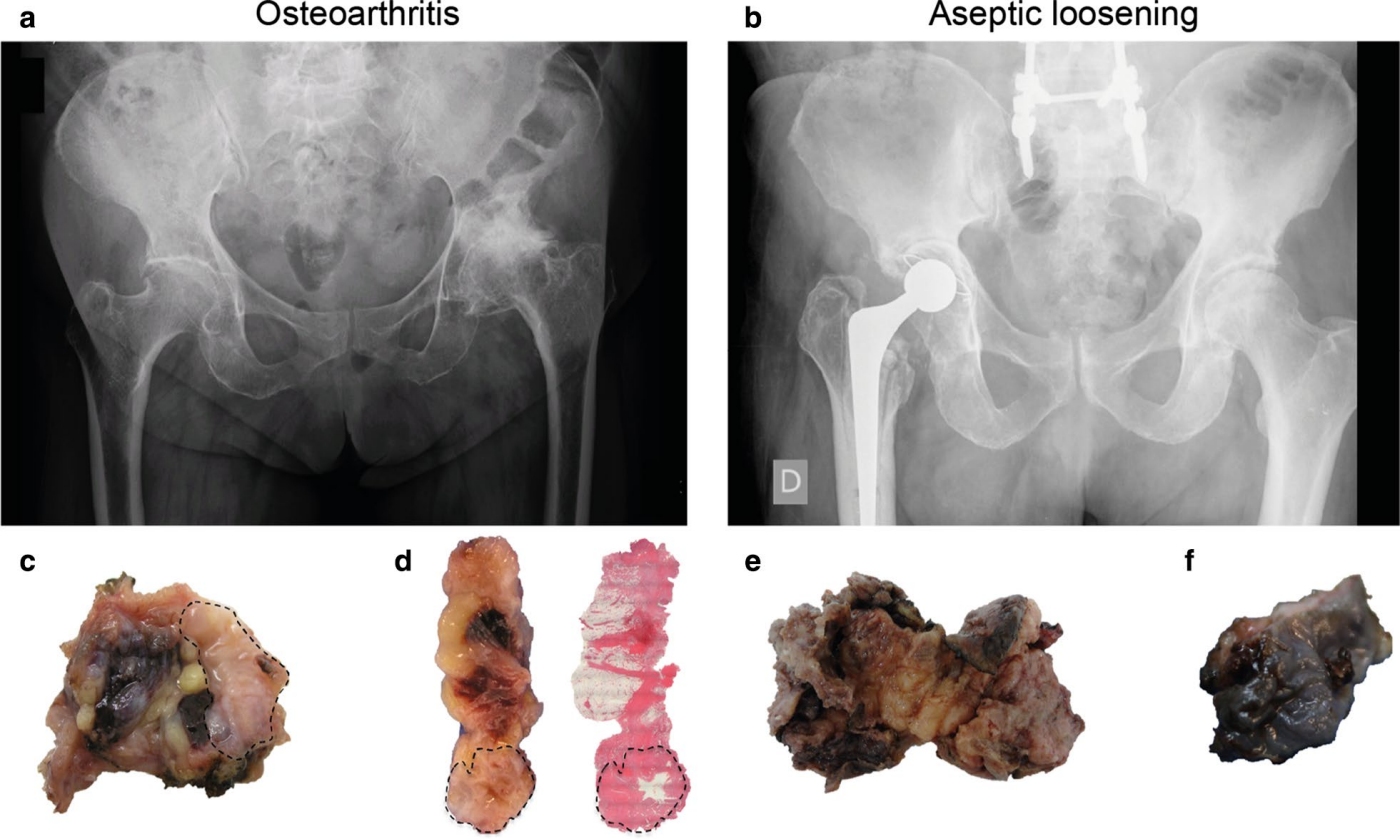
Radiological and macroscopic tissue features of OA, AL and metallosis. **a** Anteroposterior X-rays showing OA of the hip. **b** Failed metal-on-polyethylene total hip joint due to AL. **c**, **d** Macroscopic images of OA synovial tissue collected at primary hip replacement. **e** Synovial membrane-like interface tissue retrieved at hip revision surgery due to AL. **f** Metallic debris accumulation in synovial membrane-like tissue from AL patient with metallosis

In this study, 15 patients underwent primary hip replacement due to OA (Fig. 1a) and twenty hip implants were revised and their acetabular defects classified by X-ray imaging (Fig. 1b). Macroscopically, the collected synovial tissues at primary and revision surgeries were morphologically distinct. Synovial surface was white and with diffuse papillary architecture (dashed black line, Fig. 1c, d) whereas synovial membrane-like interface tissues were highly fibrotic (Fig. 1e) or with greyish appearance (Fig. 1f), in case of metallosis.

**Fig. 2:**
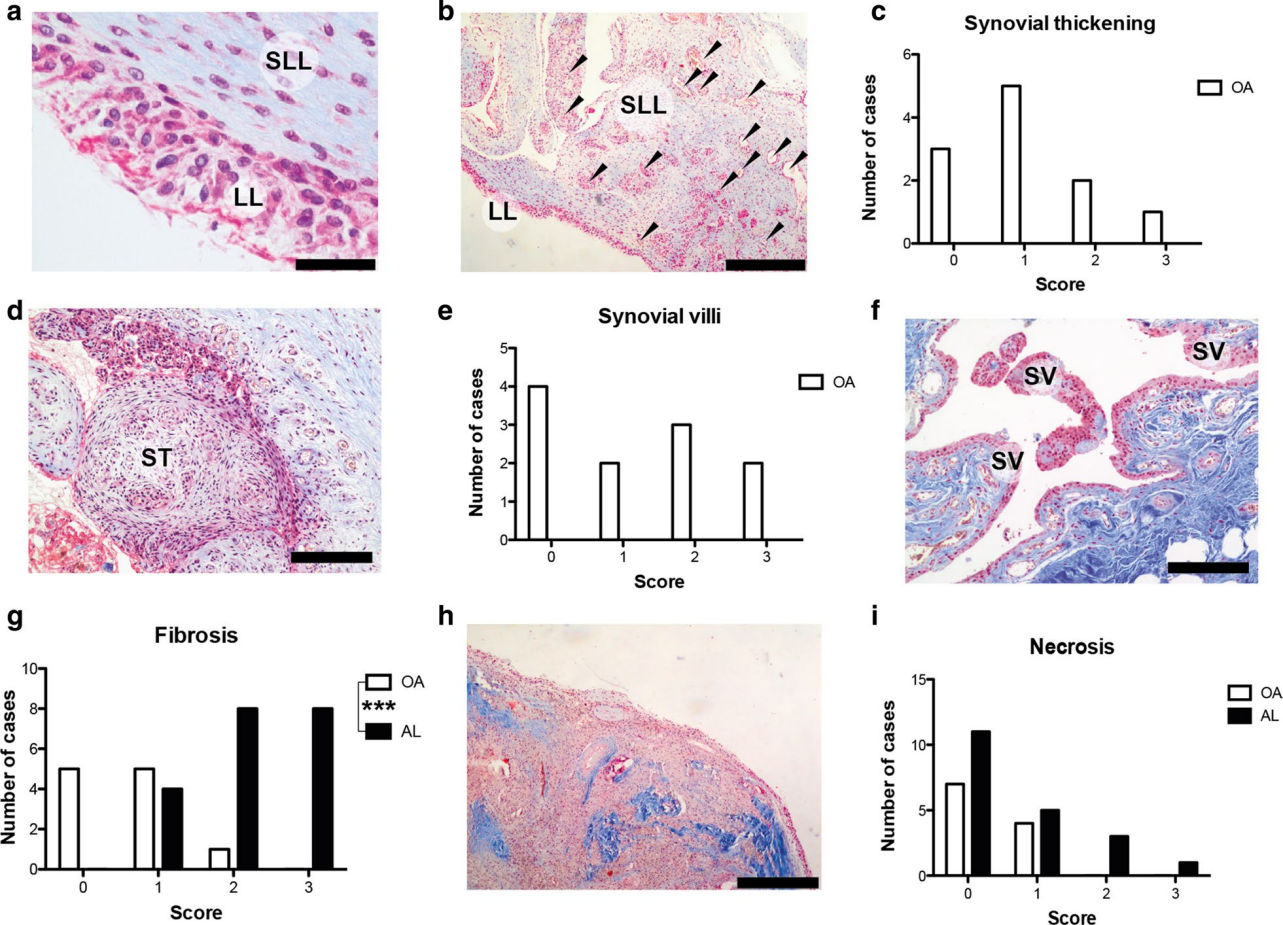
Histological evaluation of tissues organization. **a** OA synovial tissue is composed by different cell types and matrixes organized in synovial lining layer (LL) and sublining layer (SLL). **b** Dense net of blood vessels (*black arrows*) was found in SLL together with loose connective tissue (*blue stain*). **c** Histological grading for synovial membrane thickening in OA. **d** Synovial membrane thickening (ST) with reactive neo vascularization. **e** Histological grading for synovial villous expansion in OA. **f** OA synovial membrane presenting villous hypertrophy (SV). **g** Histological grading for tissue fibrosis. **h** Synovial membrane-like interface tissue showing fibrosis characterized by intense deposition of collagen fibers (*blue stain*). **i** Histological grading for tissue necrosis. Masson’s trichrome (**a**, **b**, **d**, **f**, **h**) staining. *Scale bars* correspond to 500 μm (**b**, **f**, **h**), 200 μm (**d**) and 50 μm (**a**). Semi-quantitative histological evaluation of tissue architecture was performed in specimens retrieved from 11/15 OA and 19/20 AL patients. ***p < 0.001. Chi square test was used to compare OA and AL groups

Semi-quantitative analysis of tissue organization and immune cell distribution in OA synovial tissue and aseptic interface tissues showed distinct local profiles. OA synovial membranes displayed a bicellular lining layer (LL) and a sublining layer (SLL) (Fig. 2a) enriched in blood vessels (black arrows) and collagen (Fig. 2b), as well as structural changes induced by synovial inflammation, such as synovial hyperplasia (Fig. 2c, d) and increased number of villi (Fig. 2e, f). OA patients presented at least one sign of synovial inflammation (thickening or villi) but their magnitude was variable among patients (Fig. 2c, e). Synovial membrane-like interface tissues presented fibrotic stroma with increased collagen deposition (Fig. 2g, h) than OA synovial tissues (p = 0.001) and with some necrotic regions (Fig. 2i).

**Fig. 3:**
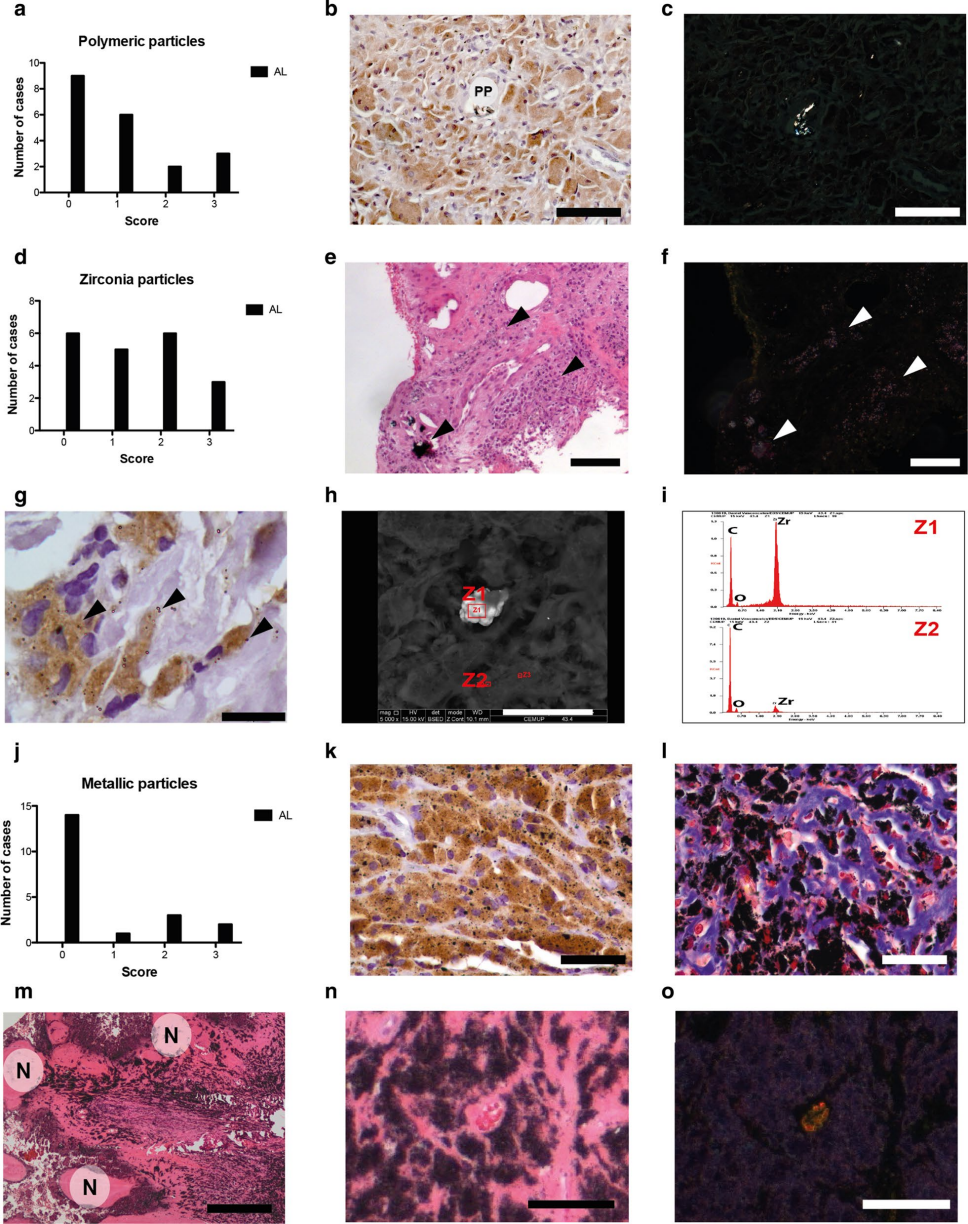
Prosthetic debris accumulation in synovial membrane-like tissues. **a** Histological grading for accumulation of polymeric particles (PP) in aseptic interface membranes. **b** PP surrounded by macrophages (*brown cells*). **c** Same section of (H) under polarized light showing birefringent PP. **d** Histological grading for deposition of ZrO_2_ particles in synovial membrane-like interface tissues. **e** H&E image of aseptic interface membrane with intense deposition of ZrO_2_ particles (*black arrows*) using conventional light microscopy. **f** Same tissue region observed using Ph3 filter with white and bright particles corresponding to ZrO_2_ debris (*white arrows*). **g** Macrophages (*brown cells*) phagocytizing ZrO_2_ debris (*black arrows*). **h** SEM image of synovial membrane-like interface tissues containing clusters (Z1) and sole (Z2) ZrO_2_ nanoparticles. **i** EDS analysis confirming the elemental composition of ZrO_2_ nanoparticles. **j** Histological grading for entrapment of metallic particles in tissues. **k** Macrophages (*brown cells*, CD68 + cells) with phagocytized metallic particles. **l** Metallic particles co-localized with macrophages and high deposition of collagen (*blue stain*). **m** Necrosis (*N*) in regions of aseptic interface membranes with massive accumulation of metallic particles. **n** Tissue from an AL patient with metallosis showing metallic particles and erythrocytes. **o** Same tissue section shwoing bright metallic particles and erythrocytes but exhibited under Ph3 filter violet and red colors, respectively. H&E staining (**e**, **m**, **n**), polarized light (**c**), Ph3 filter (**f**, **o**), Masson’s trichrome (**l**) immunohistochemistry (**b**, **g**, **k**), SEM imaging (**h**) and EDS spectra (**i**). *Scale bars* correspond to 500 μm (**e**, **m**), 50 μm (**b**, **k**, **l**) and 20 μm (**g**, **n**, **o**). Semi-quantitative histological evaluation was performed in tissues retrieved from 19/20 AL patients

The accumulation of prosthetic debris in synovial membrane-like interface tissues was semi-quantified. Polymeric particles were just detected in 11/20 analyzed aseptic interface tissues (Fig. 3a) after tissue analysis under polarized light (Fig. 3b, c). No significant difference was found between AL patients with cemented and uncemented MoP bearings regarding the amount of polymeric particles entrapped in synovial membrane-like tissues. Zirconia particles (ZrO_2_) were observed in almost all synovial membrane-like interface tissues retrieved from patients with loose cemented prostheses (Fig. 3d–f) and mainly phagocytized by macrophages or multinucleated giant cells (Fig. 3g). Ph3 contrast filter eased the detection of ZrO_2_ particles (Fig. 3e, f). White color particles under Ph3 filter were confirmed to be ZrO_2_ and to be organized in clusters of nanoparticles by SEM/EDS (Fig. 3h, i). Intense deposition of metallic particles was just found in the four cases of metallosis that were patients with uncemented metal-back acetabular cups (Fig. 3j). Aseptic interface membranes with metallosis presented high deposition of metallic particles with concomitant macrophage infiltration (Fig. 3k), tissue fibrosis (Fig. 3l) and necrosis (Fig. 3m). Under Ph3 filter, metallic nanoparticles and haemoglobin presented violet and red color respectively, which allow distinguishing them from ZrO_2_ particles (Fig. 3n, o).

**Fig. 4:**
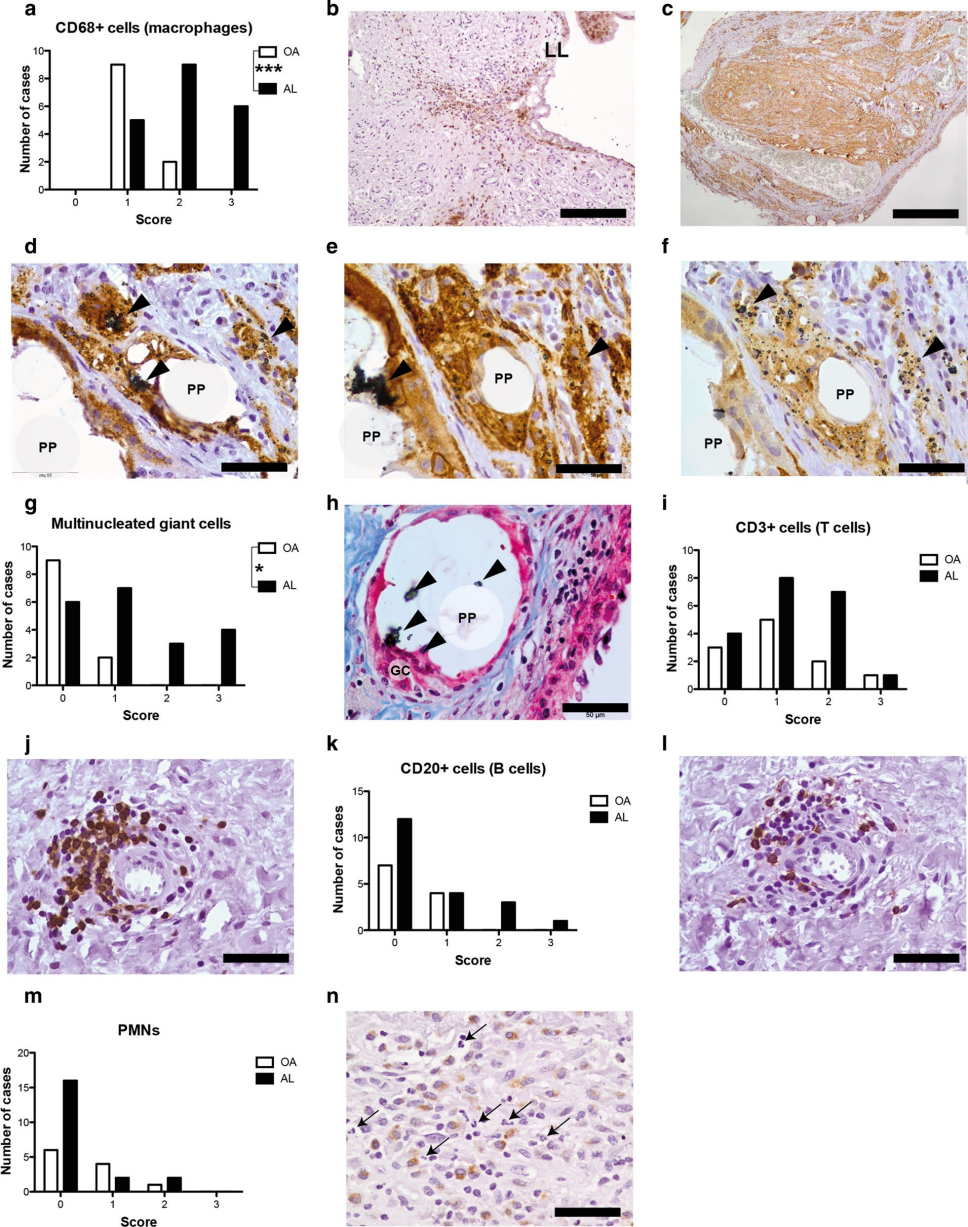
Immune cells distribution in OA synovial tissues and aseptic interface membranes. **a** Histological grading for macrophage (CD68+ cells) infiltration in tissues. **b** In OA, macrophages (*brown cells*) were almost found at LL. **c** Intense macrophage (*brown cells*) infiltration was detected in synovial membrane-like interface tissues. **d** In aseptic interface tissues, macrophages (*brown cells*) were often found surrounding or phagocytosing prosthetic debris such as polymeric particles (PP) and ZrO_2_ particles (black arrows). **e** HLA-DR + cells—an M1 macrophage marker. **f** CD163+ cells—an M2 macrophage marker. **g** Histological grading for multinucleated giant cells in tissues **h** Multinucleated giant cells (GC) phagocytosing a big PP particle with ZrO_2_ particles (*black arrows*) entrapped inside. **i** Histological grading for T cells (CD3+ cells). **j** Perivascular T cells (*brown cells*) clusters in OA synovial tissue. **k** Histological grading for B cells (CD20+ cells). **l** B cells (*brown cells*) in lymphocyte aggregates around blood vessels but in lower number than T cells. **m** Histological grading for polymorphonucleated cells (PMNs). **n** Increased number of PMNs (*black arrows*) in synovial membrane-like tissue with macrophages (*brown cells*, CD68+ cells). Masson’s trichrome staining (**h**) and immunohistochemistry (**b**, **c**, **d**, **e**, **f**, **j**, **l**, **n**). *Scale bars* correspond to 500 μm (**b**, **c**) and 50 μm (**d**, **e**, **f**, **h**, **j**, **l**, **n**). Semi-quantitative histological evaluation was performed in synovial tissues retrieved from 11/15 OA and 19/20 AL patients. *p < 0.05, ***p < 0.001. Chi square test was used to compare OA and AL groups

Immune cell distribution was studied in tissues from AL and OA patients. In AL, macrophages (CD68^+^ cells) were more abundant than in OA (p = 0.007; Fig. 4a). While macrophages were often confined to lining layer in OA patients (Fig. 4b), signifficant macrophage infiltration was found in synovial membrane-like interface tissues (Fig. 4c). In these tissues, co-localization between polymeric particles and macrophages was often found (Fig. 4d) and a similar pattern was observed in AL patients with cemented and uncemented MoP bearings. In AL patients, macrophages were the most prevalent immune cell population, even in metallosis cases, and in overall highly express M1 (HLA-DR; Fig. 3e) and M2 (CD163; Fig. 3f) markers. Multinucleated giant cells in foreign body reaction setting were mostly found in synovial membrane-like interface tissues (p = 0.037, Fig. 4g) surrounding big polymeric particles, likely PMMA, with ZrO_2_ particles entrapped inside (Fig. 4h). T cells (CD3^+^ cells) (Fig. 4j), and B cells (CD20^+^ cells; Fig. 4l) in lower number, could be detected in the majority of tissues collected from AL and OA patients (Fig. 4i, k), including AL patients with uncemented implants or signs of metallosis. Moreover, the number of PMNs was also low in both aseptic interface membranes and OA synovial tissues. Overall, the immune cell populations addressed, namely macrophages, T cells, B cells and PMNs, in this study were most located in the vicinity of synovial membrane in OA patients, while in AL patients these cells were identified throughout the aseptic interface membranes.

### Local innervation in AL and OA patients

**Fig. 5:**
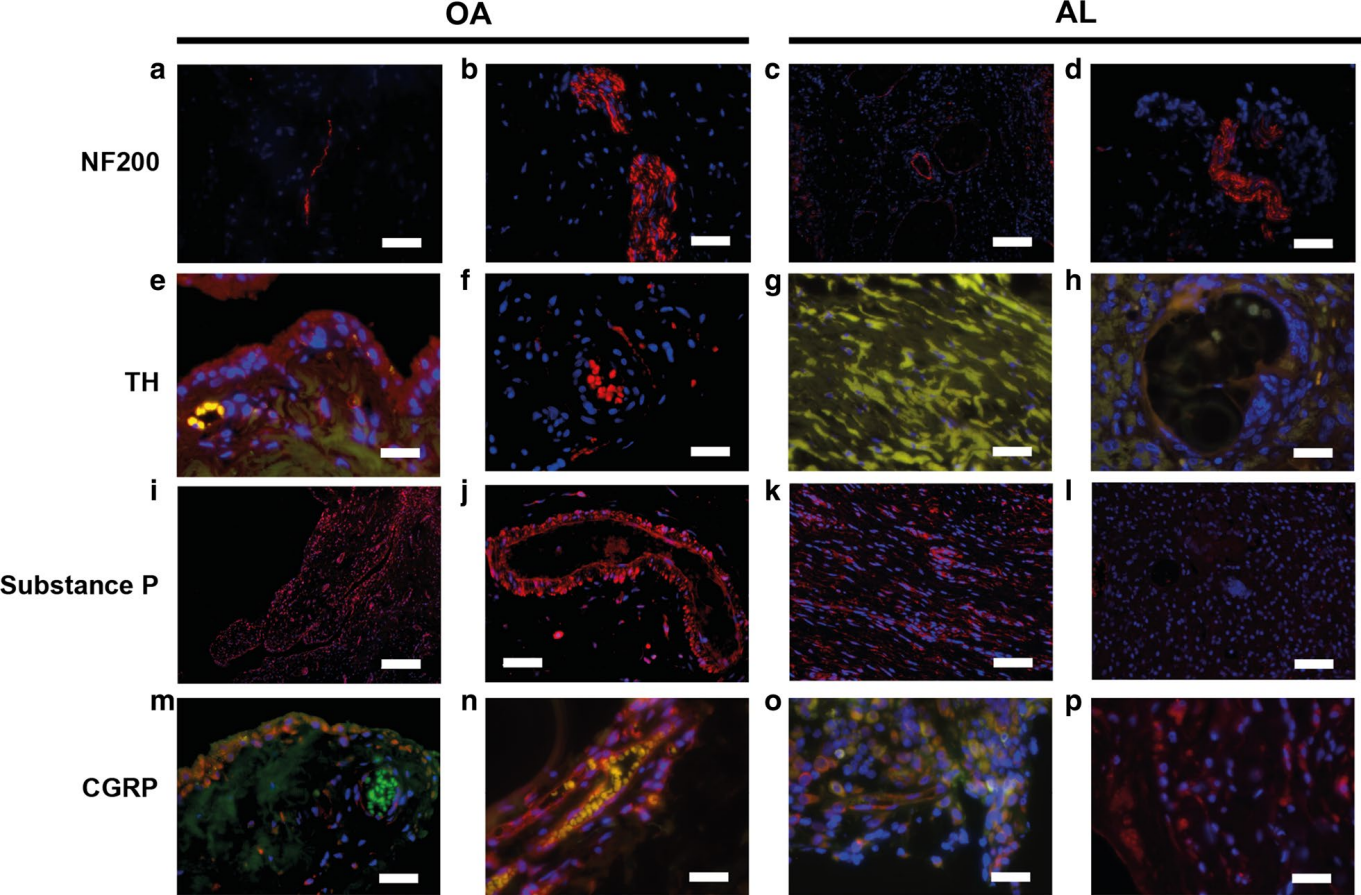
Local tissue innervation in OA and AL patients. **a**–**d** NF200+ fibers (*red*) as sole fiber or organized in neurome-like structures in OA synovial tissues as well as in aseptic interface membranes. **e** Synovial cells expressing TH (*red*). **f** TH+ fiber surround a blood vessel. **g**, **h** Nor fibrotic nor reactive tisue with giant cells presented positive labelling for TH. **i** SP+ cells in OA synovium membrane. **j** SP+ fibers and SP+ cells surrounding a blood vessel located at subintimia of OA synovial tissue. **k**, **l** In AL patients, SP+ fibers were just found in fibrotic regions. **m** CGRP+ cells in synovial membrane. **n** CGRP+ fibers along a blood vessel in OA synovial tissue. **o**, **p** Some cells expressing CGRP in aspetic interface tissue. *red* = NF200+ or TH+ or SP+ or CGRP+ , *blue* = cell nuclei, *green* = autofluorescence) *Scale bars* correspond to 50 μm (**a**, **b**, **d**, **f**, **g**, **h**, **i**, **j**, **k**, **l**, **m**, **n**, **o**, **p**), 200 μm (**e**) and 100 μm (**c**)

Myelinated nerve fibers identified by NF200 immunoreactivity were detected in both synovial membrane-like interface tissues and OA synovial tissues. They were presented as single fibers (Fig. 5a, c) and were preferentially arranged around blood vessels (Fig. 5c) particularly in tissue regions of reactive vascularization induced by immune responses underlying AL and OA. Alternatively, nerve fibers were also found in neurome-like structures (Fig. 5b, d) in both AL and OA patients. Sensory and sympathetic innervation, as seen by immunohistochemical markers of sensory nerve-associated peptides (SP and CGRP) and catecholaminergic marker of sympathetic neurons (TH), showed different pattern between OA patients and AL patients. TH immunoreactive nerve fibers were observed in OA synovial tissues (Fig. 5f) but not in synovial membrane-like interface tissues (Fig. 5g, h). SP and CGRP were found both in OA synovial tissues and synovial membrane-like interface tissues but showed different pattern. In OA synovial tissues, nerve fibers immunoreactive to SP and SGRP were observed mainly around blood vessels in the vicinity of synovial membrane (Fig. 5j, n). In addition, a high number of cells in the OA synovial membrane also stained for TH, SP and CGRP (Fig. 5e, i, m). In synovial membrane-like interface tissues, the expression of SP and CGRP was also found in both nerve fibers and cells but with a broad distribution throughout the tissue (Fig. 5k, l, o, p).

### Local gene expression profile

**Fig. 6:**
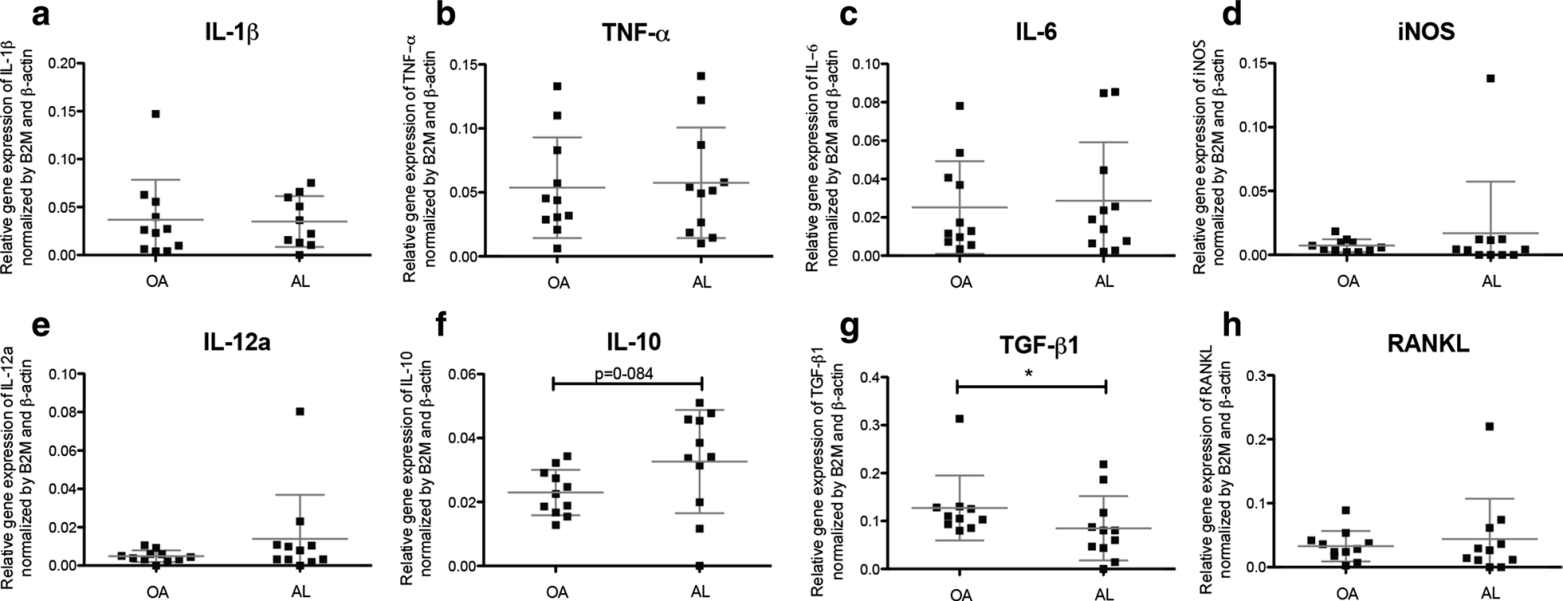
Gene expression profiles of cytokines in aseptic interface membrane and OA synovial tissues. Relative expression levels of genes of interest determined through qRT-PCR and normalized by two reference genes, β-actin and B2 M: **a** IL-1β. **b** TNF-α. **c** IL-6. **d** iNOS. **e** IL-12a. **f** IL-10. **g** TGF-β1 **h** RANKL. Data of 10/15 OA patients and 11/20 AL patients are shown. Significant difference of TGF-β1 expression between AL and OA groups (*p < 0.05). Mann–Whitney test was utilized to compare and analyze the obtainded data

The expression levels of pro-inflammatory cytokines tumor necrosis factor-α (TNF-α), interleukin-1β (IL-1β) and IL-6 were similar in aseptic interface tissues and OA synovial tissues (Fig. 6a–c). Inducible nitric oxide synthase (iNOS) and IL-12a expression levels were found to be low when compared with the TNF-α, IL-1β and IL-6 mRNA levels detected in AL and OA groups (Fig. 6d, e). Interestingly, the anti-inflammatory cytokine IL-10 presented a tendency (p = 0.084) to be higher expressed in synovial membrane-like tissues than in OA synovial tissues (Fig. 6f). Two genes involved in bone remodeling were evaluated: TGF-β1 and receptor activator of nuclear factor kappa-B ligand (RANKL). The mRNA levels of TGF-β1 were significantly reduced (p = 0.038) in aseptic interface tissues when compared to OA synovial tissues (Fig. 6g). However, no differences between AL and OA patients were verified for RANKL (Fig. 6h) and no correlation was found between mRNA levels of RANKL and the bone defect type.

### Local TGF-β1 expression

**Fig. 7:**
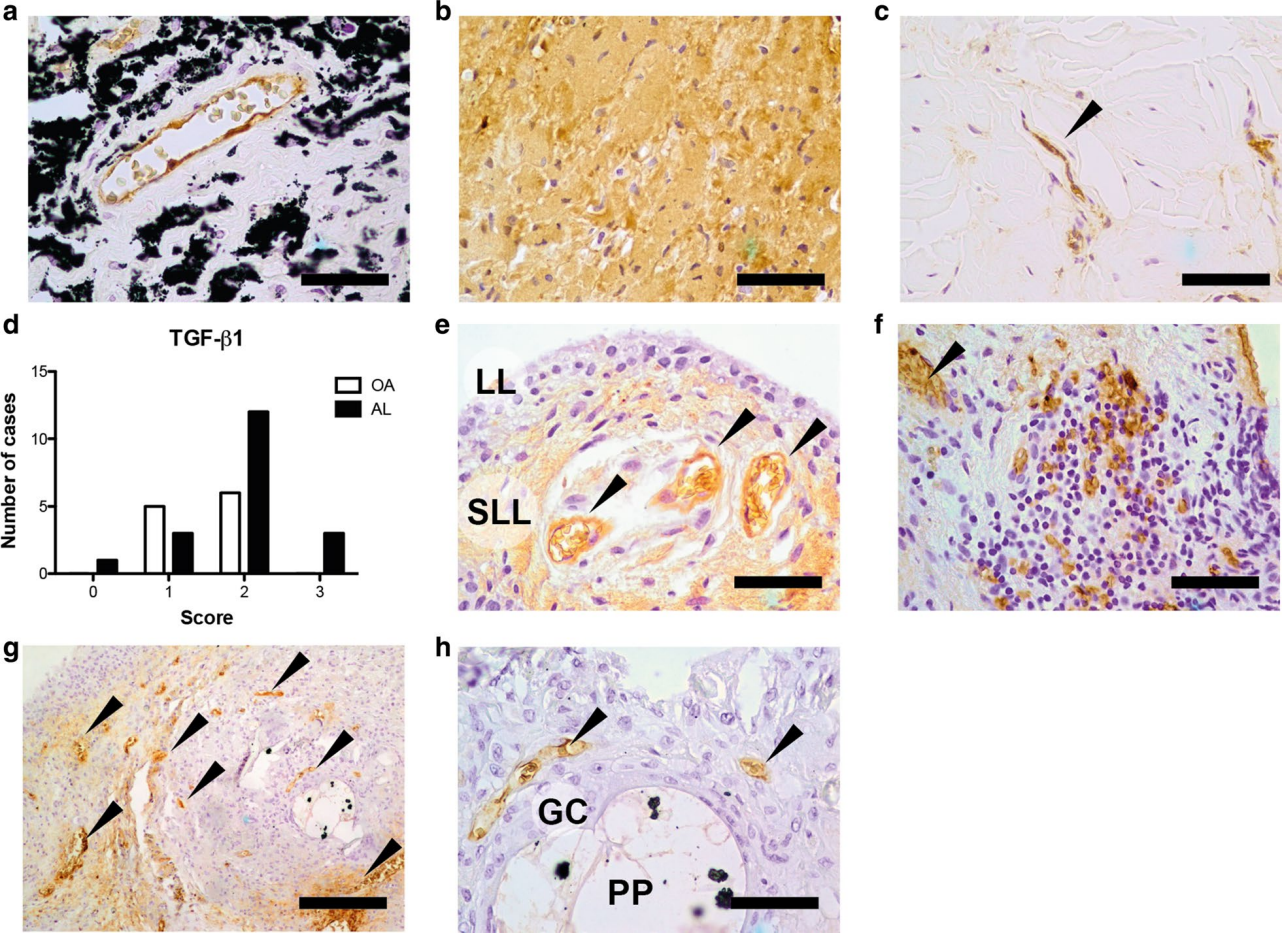
TGF-β1 expression in synovial membrane-like interface tissues and OA synovial tissues. **a** TGF-β1+ endothelial cells in aseptic interface tissue with metallosis. **b** TGF-β1+ macrophages in synovial membrane-like interface tissue. **c** TGF-β1+ fibroblast in aseptic interface membrane. **d** Number of OA and AL patients classified with score from 0 to 3 regarding the presence of TGF-β1 in tissue. **e** OA synovial membrane with positive labelling at sublining layer and endothelium (*black arrows*). **f** Lymphocyte aggregate in OA synovial tissue with positive cells for TGF-β1. **g** Aseptic interface membrane presenting heterogenous TGF-β1 labelling. **h** Multinucleated giant cell (GC) phagocytosing a polymeric particle (PP) with ZrO_2_ particles inside (*black clusters*) with TGF-β1+ endothelial cells in the vicinity. Scale bars correspond to 500 μm (**g**) and 50 μm (**a**, **b**, **c**, **e**, **f**, **h**). Data was collected for 11/15 OA patients and for 19/20 AL patients. Chi square test was used to compare OA and AL groups

In both AL and OA patients, TGF-β1 was detected in blood vessels endothelium cells (Fig. 7a), macrophages (Fig. 7b) and fibroblasts (Fig. 7c). Interestingly, differences in the pattern of TGF-β1 expression were found between AL patients and OA. Aseptic interface tissues presented a trend (p = 0.1672) toward increased number of regions expressing TGF-β1 (Fig. 7d). In OA synovial tissues, TGF-β1 was present in the sublining layer of synovial membrane (Fig. 7e), in aggregates of lymphocytes (Fig. 7F) and blood vessels. In synovial membrane-like interface tissues, the distribution of TGF-β1-positive regions was heterogeneous (Fig. 7g, h).

### Immune cells proportions and TGF-β1 concentration in blood

**Fig. 8:**
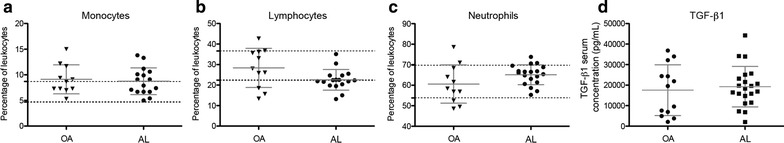
Pre-operative evaluation of immune populations and TGF-β1 in blood. Data regarding the percentage of the following immune population in leukocytes count: **a** Monocytes, **b** Lymphocytes and **c** Neutrophils. Data was collected for 14/15 OA patients and for 17/20 AL. *Dashed lines* represent minimum and maximum reference values. **d** TGF-β1 serum concentration was determined for 13/15 OA patients and for all 20 AL patients

The preoperative leukograms were analyzed and the concentration of TGF-β1 determined in plasma of both AL and OA patients. The percentage of circulating monocytes in both groups tended to be similar (Fig. 8a). Interestingly, the percentage of lymphocytes seemed to be low in AL patients but within the reference interval in OA group (Fig. 8b). Neutrophils did not present significant alterations compared with the reference values (Fig. 8c). Remarkably, the levels of TGF-β1 in serum were similar in both groups (Fig. 8d).

## Discussion

**Fig. 9:**
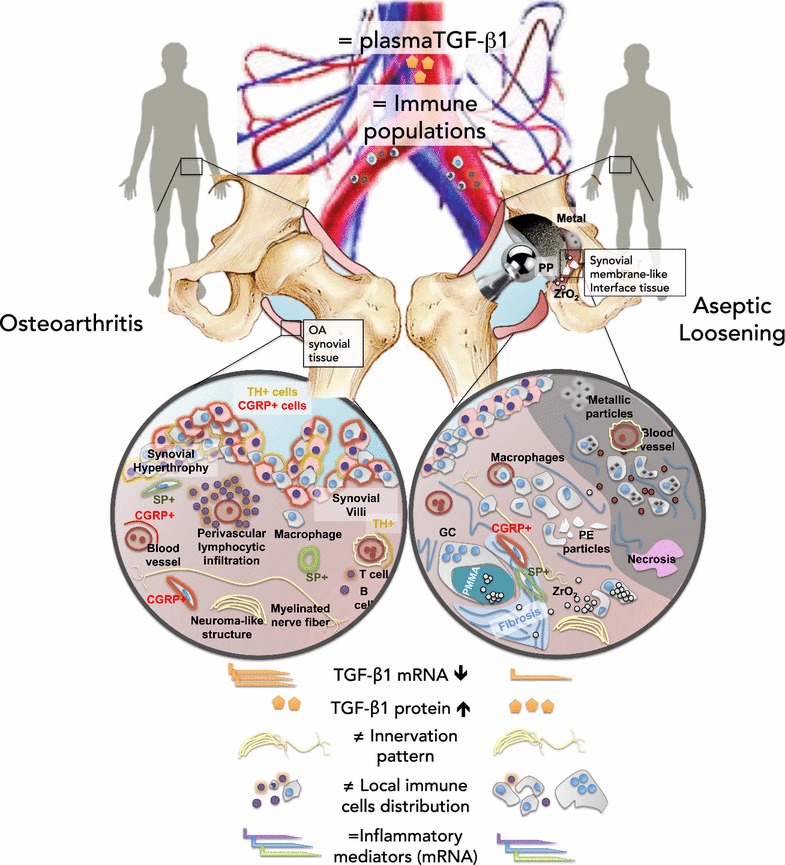
Overview of hip microenvironment in OA and AL

AL and OA differences rely on tissue architecture, immune cell distribution, local TGF-β1 expression as well as sensory and sympathetic synovial innervation. On the other hand, both pathologies share identical inflammatory mediators mRNA profiles and similar TGF-β1 concentrations in serum, as summarized in Fig. 9.

Synovial membrane-like interface tissues, present in the vicinity of loose prostheses, showed a macrophage-driven chronic inflammation. This immune response is likely influenced by both OA inflammatory background and the presence of prosthetic debris, leading to significant changes of local TGF-β1 expression but not systemically.

Distinct tissue organization and immune cell distribution were found in the tissues retrieved from the hip joint of AL and OA patients. OA synovial tissues presented signs of synovial inflammation (synovitis), such as synovial hyperplasia and villous hypertrophy, while aseptic interface membranes, formed after primary hip replacement, exhibited a particle-driven chronic inflammation. Intense infiltration of macrophages (CD68^+^ cells) was observed in AL patients in comparison to OA synovial tissues. In synovial membrane-like interface tissues retrieved from AL patients, macrophages were the predominant immune cell type and were involved in the phagocytosis of small particles (<10 μm) or encapsulating bigger polymeric particles as multinucleated giant cells, in line with previous works about the role of macrophages on AL [28]. Although PE particles, released by MoP prostheses, have been pointed out as AL catalyzers [29, 30], other types of particles may play a role on the pro-inflammatory microenvironment underlying osteolysis. Large amounts of metallic particles were observed in four out nine AL patients due to impingement of metallic components of uncemented MoP bearings.

Ceramic ZrO_2_ particles, incorporated in bone cements for implants fixation as radiopacifier agent, were found in synovial membrane-like interface tissues from all the patients with cemented prostheses. Light microscopy with Ph3 contrast filter and SEM/EDS analysis revealed that ZrO_2_ gradually migrate from cement particles (PMMA) to other regions of aseptic interface tissues in high number, namely after being phagocytized by macrophages. ZrO_2_ particles were shown to be moderately toxic, activate macrophages, promote the expression of pro-inflammatory cytokines (TNF-α) and induce osteolysis in vivo [31, 32]. Despite reported not toxic as metallic particles or studied as polymeric debris [12, 33, 34], ZrO_2_ particles should not be neglected, as they are numerous, common and nano-sized with potential to unbalance inflammation towards osteolysis. It has been reported that particle-induced response is prone to drive macrophages towards M1 phenotype and that M1:M2 ratio was higher in synovial membrane-like interface tissue than in OA synovial tissues [35, 36]. However, macrophage polarization in joint tissues remains controversial. In this study, the density of polymeric, metallic or ZrO_2_ particles on aseptic interface tissues did not lead to local preferential macrophage polarization in M1 pro-inflammatory (HLA-DR^+^) or M2 pro-regenerative phenotypes (CD163^+^) as both receptors were similarly expressed on macrophages. In addition to the involvement of macrophages and lymphocytes in local inflammatory response, preoperative leukograms suggested monocyte expansion and contraction of lymphoid population in AL group.

Cytokines expression profile was found similar in synovial membrane-like interface tissues and OA synovial tissues, despite their distinct tissue architecture and immune cell distribution. The response of macrophages to prosthetic debris is believed to induce the production of the pro-inflammatory markers such as TNF-α, IL-1β, IL-6, IL-12a, and iNOS [28, 34, 37]. However those genes revealed similar mRNA levels in AL and OA patients, which is in line with comparable studies [26, 30, 38]. The changes in cytokine expression induced by AL seem to be controversial [25]. Reduced mRNA levels of IL-6 have also been described in aseptic interface membranes [26] while IL-6 was found significantly increase in synovial fluid of AL patients [25, 30]. A trend for higher mRNA levels of the anti-inflammatory cytokine IL-10 was observed in synovial membrane-like interface tissues in comparison to OA synovial tissues (p = 0.084). This result corroborates with other findings showing significant increase of IL10 protein levels in synovial fluid and interface tissues of AL patients [25, 26, 30]. The up-regulation of IL-10 in aseptic interface tissues may constitute an attempt to balance the pro-inflammatory microenvironment induced by prosthetic debris [39, 40].

Although RANKL is involved in osteoclastogenesis and osteolysis, similar mRNA levels were found in AL and OA patients, in agreement with other authors [26, 30, 38, 41]. In overall, identical pattern regarding the expression of cytokines was found in AL and OA patients. In the context of AL, no significant differences were registered between polyethylene-drive and metallosis cases as previously shown [42]. The expression of TGF-β1 mRNA in synovial membrane-like interface tissues was found significantly lower than in OA synovial tissues but local TGF-β1 immunostaining suggested increased expression of this protein in AL patients in comparison to OA patients. These findings corroborate the results presented by other authors that have also found increased TGF-β1 mRNA levels in OA cartilage [9, 43, 44] and augmented TGF-β1 expression in synovial membrane-like interface tissues [45]. In both AL and OA, TGF-β1^+^ labeling was prominent in sites where the inflammation is occurring and mostly expressed in macrophages, fibroblasts and endothelium cells. However, while in OA the TGF-β1^+^ cells were found in the region of synovial membrane, in AL, the distribution was more heterogeneous and throughout different tissue regions. These results suggest a possible association between TGF-β1 and the immune responses that underlie AL and OA. Despite a previous report indicates that in OA pathogenesis, TGF-β1 might have a pro-inflammatory effect by inducing fibroblasts to express TNF-α and IL-1β [46], its involvement in inflammatory response is not fully understood. Although the involvement of TGF-β1 in fibrosis is widely described, this growth factor may have dual effects on arthritic diseases [47]. Both immune cells and fibroblasts are part of the complex microenvironments that underlie AL and OA and in which TGF-β1 may have different effects depending on levels of other inflammatory mediators. Additionally, in the context of particle-induced immune response, TGF-β1 has a conjoint role with other factors on bone remodeling [12].

Moreover, previous studies demonstrated that serum TGF-β1 has not predictive value to assess OA incidence and progression [48, 49]. In our study, despite the difference in tissues regarding TGF-β1, AL and OA patients presented similar concentrations of TGF-β1 in serum.

The crosstalk between inflammation and innervation has been widely investigated in several joint-related disorders but not in presence of prostheses [50, 51]. Previous studies have reported sensory and sympathetic innervation in joints diseases namely OA and rheumatoid arthritis (RA), where an inflammatory response is taken place within synovial tissues or in the vicinity of the articulation. Our study demonstrated for the first time distinct pattern of sensory and sympathetic innervation in AL patients characterized by a loss of sympathetic nerve fibers when compared to OA patients. This differential profile has been also described in a comparative study showing lack of sympathetic innervation in RA patients while in OA patients this does not occur [52]. The authors suggested that the reduction or absence of sympathetic innervation might be a consequence of the initial synovial inflammation, probably involving nerve repellent molecules [53], and a key factor to its maintenance in RA. In AL, a sustained chronic inflammatory response is maintained by the presence of prosthetic debris, primarily generated at the bearing surface of hip prostheses. Therefore, our findings, supported by those described in RA, strongly indicate a close correlation between sympathetic innervation pattern and the degree of the inflammatory response. A previous study described the appearance of TH^+^ cells in the collagen-induced arthritis in mice and hypothesized that the presence of those cells might be a compensatory mechanism to the deprivation of sympathetic neurotransmitters in the joint [54]. In AL patients, TH^+^ catecholamine-producing cells were not detected in the synovial membrane-like interface, suggesting total uncoupling of local joint inflammation from the sympathetic activity (nerve fibers and cells).

Several animal and human studies have addressed the pattern of the sensory innervation in arthritis, namely in OA, and reported a significant re-organization of the sensory nerve fibers characterized by an alteration in the morphology, density and sprouting into areas of the joint that are normally poorly innervated [21]. In this study we showed that there is also alteration in the sensorial innervation of synovial membrane-like interface tissues in AL patients with a notable distinct tissue distribution when compared to OA. SP and CGRP immunoreactive nerve fibers were observed in subintima regions (outer layer) mainly around blood vessels while in AL they were distributed throughout synovial membrane-like interface tissues. Of note, SP^+^ and CGRP^+^ cells were also found in these tissues as an additional source of these neuropeptides. Although, SP and CGRP are recognized as neuro-inflammatory modulators in arthritic joints, their functional role in the inflammatory response associated to prosthetic debris as well as in the reorganization of sensory and sympathetic nerve fibers in inflamed joint need to be clarified.

We acknowledge certain limitations in this study. To study the differences between AL and OA, thirty-five patients were included in our study, an average number in the field [40]. OA synovial tissues and synovial membrane-like interface tissues were retrieved by the same surgeon to minimize location bias. The histological semi-quantification of accumulation of prosthetic debris in aseptic interface tissues was limited to micro-sized particles, or clusters of nanoparticles, although tissues have been previously evaluated by SEM. Gene expression analysis was successfully performed in eleven out of twenty AL patients due to low RNA quality. Patients’ stratification concerning implant fixation and metallosis (AL group) or disease severity (OA group) was performed but no significant effect was identified. On the other hand, the interpretation of the variability between patients may not be just influenced by a specific biological response in joint region but by other factors such as patients comorbidities (e.g. diabetes mellitus and dyslipidemia), geriatric condition, or genetic susceptibility to AL [16, 55].

## Conclusions

Overall this study showed that AL and OA are two joint pathologies characterized by local immune response however with distinct tissue organization and immune cell distribution. This differential immune profile is also accompanied with changes of sensory and sympathetic innervation in hip joint. These findings highlight that the interplay between inflammation and innervation may be joint pathology-specific. Therefore, a deeper and conjoint understanding of these processes will constitute a solid base for targeted-therapies to improve hip joint lifetime and treatment.

## Additional files

10.1186/s12967-016-0950-5 Histological grading applied in semi-quantification of OA synovial tissues inflammation and tissue fibrosis, necrosis, innervation (NF200) and TGF-β1 in tissues collected from OA and AL patients.
10.1186/s12967-016-0950-5Histological grading applied in semi-quantification regarding the accumulation of prosthetic debris in synovial membrane-like interface tissues.
10.1186/s12967-016-0950-5 Histological grading applied in semi-quantification of immune cells prevalence and distribution in tissues retrieved from OA and AL patients.
