# Supplementary figures and images for: Immune response and innervation signatures in aseptic hip implant loosening
Source: J Transl Med. 2016 Jul 7;14:205. doi: 10.1186/s12967-016-0950-5 (PMC4937545; doi:10.1186/s12967-016-0950-5)

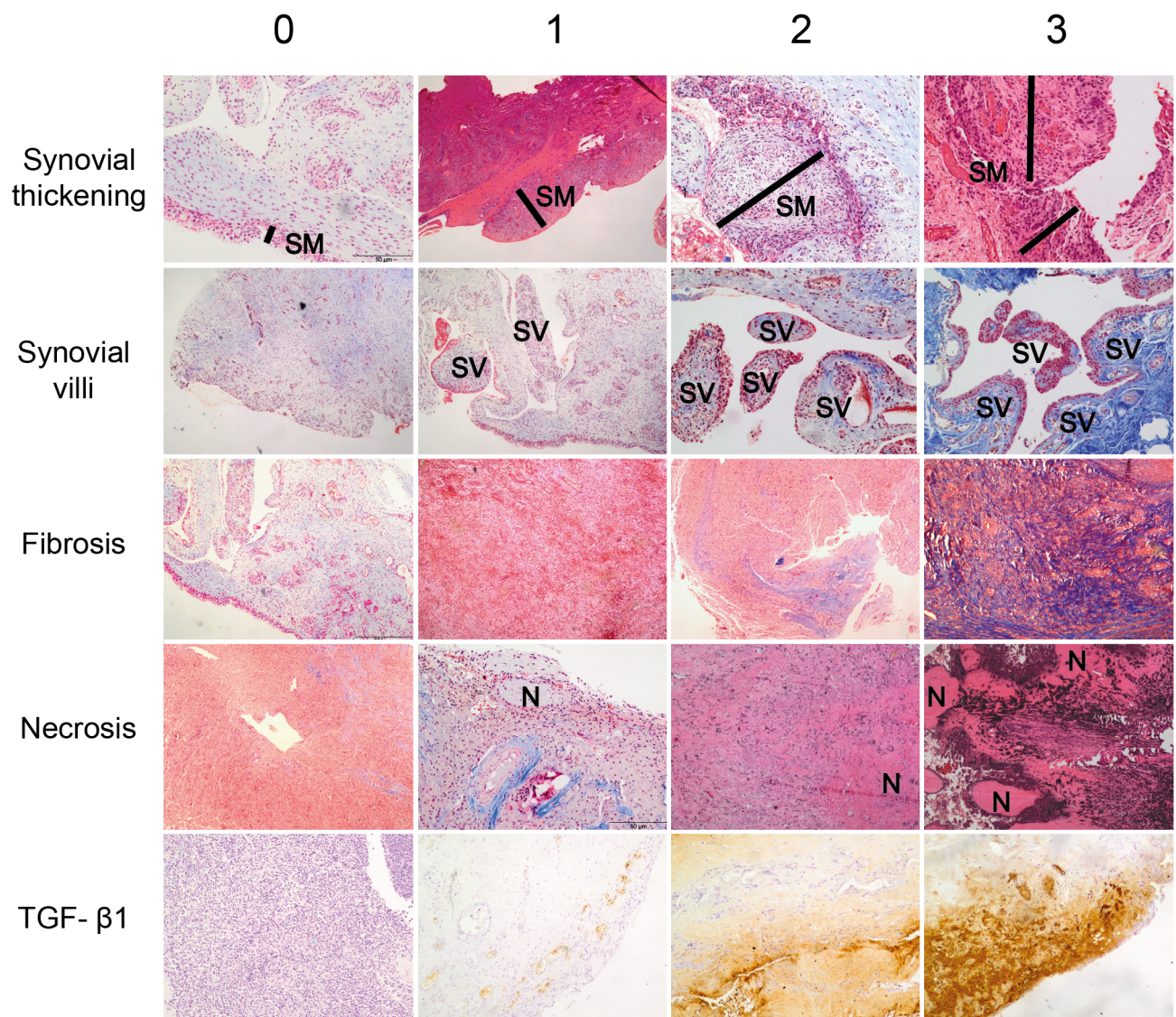

Supplement: Supplementary file 1 — 10.1186/s12967-016-0950-5 Histological grading applied in semi-quantification of OA synovial tissues inflammation and tissue fibrosis, necrosis, innervation (NF200) and TGF-β1 in tissues collected from OA and AL patients. [file 12967_2016_950_MOESM1_ESM.pdf]

1

2

3

Polymeric  
particles

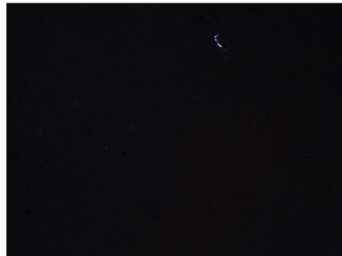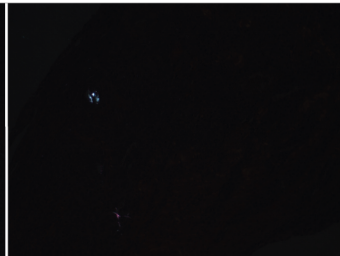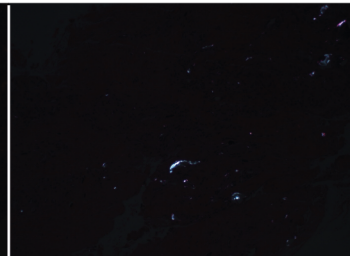

Metallic  
particles

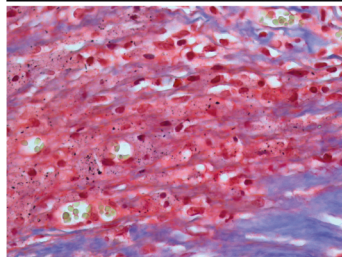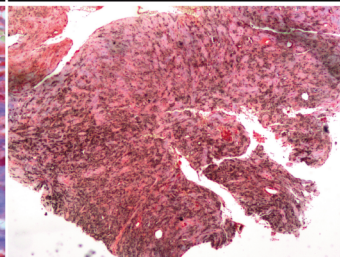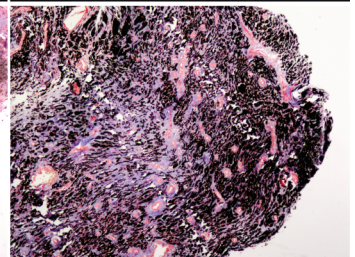

ZrO<sub>2</sub>  
(Ph3 filter)

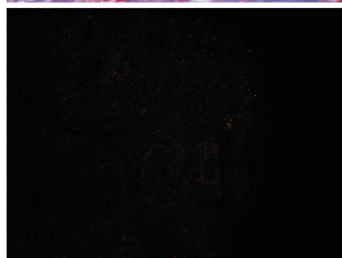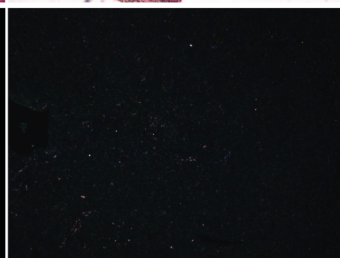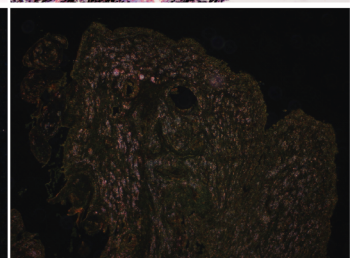

Supplement: Supplementary file 2 — 10.1186/s12967-016-0950-5Histological grading applied in semi-quantification regarding the accumulation of prosthetic debris in synovial membrane-like interface tissues. [file 12967_2016_950_MOESM2_ESM.pdf]
