# Supplementary material for: Immune response and innervation signatures in aseptic hip implant loosening
Source: J Transl Med. 2016 Jul 7;14:205. doi: 10.1186/s12967-016-0950-5 (PMC4937545; doi:10.1186/s12967-016-0950-5)
Supplement: Supplementary file 3 — 10.1186/s12967-016-0950-5 Histological grading applied in semi-quantification of immune cells prevalence and distribution in tissues retrieved from OA and AL patients. [file 12967_2016_950_MOESM3_ESM.pdf]

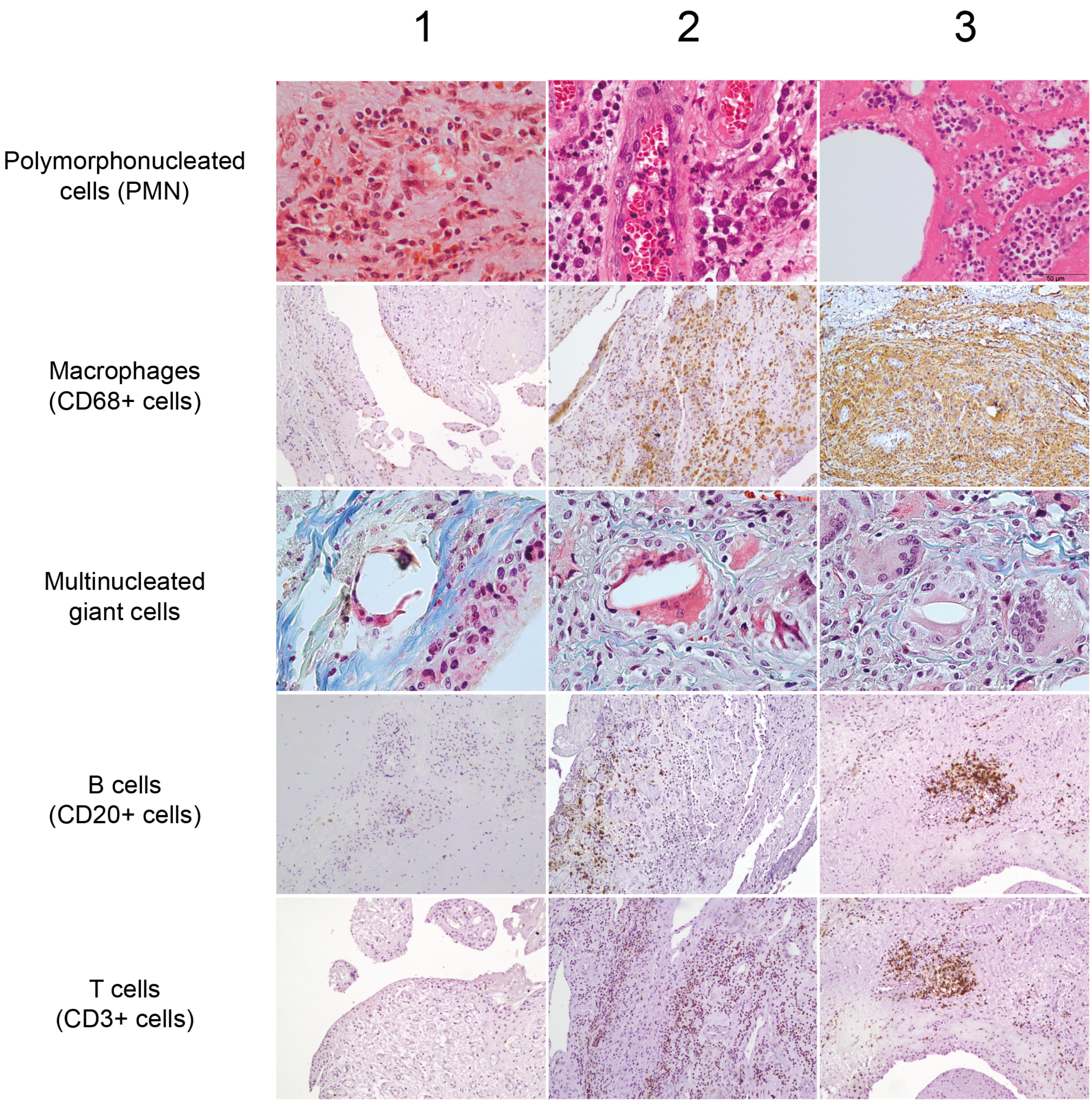
1
2
3
Polymorphonucleated cells (PMN)
Macrophages (CD68+ cells)
Multinucleated giant cells
B cells (CD20+ cells)
T cells (CD3+ cells)
